# Pain Interference Mediates the Relationship between Pain and Functioning in Pediatric Chronic Pain

**DOI:** 10.3389/fpsyg.2016.01978

**Published:** 2016-12-26

**Authors:** Rikard K. Wicksell, Marie Kanstrup, Mike K. Kemani, Linda Holmström

**Affiliations:** ^1^Behavior Medicine Pain Treatment Service, Karolinska University HospitalStockholm, Sweden; ^2^Department of Clinical Neuroscience, Karolinska InstitutetStockholm, Sweden; ^3^Neuropediatric Research Unit, Department of Women’s and Children’s Health, Karolinska InstitutetStockholm, Sweden

**Keywords:** pain, chronic, pediatric, interference, functioning, depression

## Abstract

Pediatric chronic pain is a major health problem commonly associated with impaired functioning. There is a great need for more knowledge regarding the complex interplay between demographic variables such as age and gender, pain, and functioning in pediatric chronic pain.

**Objective:** The objective of the study was to investigate if; (1) pediatric chronic pain patients with high and low levels of functioning differ in demographic variables, pain, and pain interference; (2) explore the mediating function of pain interference in the relationship between pain and functioning (i.e., depression and functional disability).

**Method:** The study includes a consecutive sample of children and adolescents referred to a tertiary pain clinic due to chronic pain (*n* = 163). Cross-sectional data was analyzed to investigate the interrelationships between variables. Analyses of indirect effects were used to assess the impact of pain interference on the relation between pain and depression.

**Results:** Findings illustrate high levels of depression, school absence and pain interference in this sample. Furthermore, pain interference mediated the relationship between pain and depression.

**Conclusion:** Thus, this study adds to the growing support of findings suggesting that functioning and pain interference should be routinely assessed in pediatric chronic pain and a central target in treatment. Particularly, these findings imply a need for interventions specifically aimed at improved functioning for patients with chronic debilitating pain.

## Introduction

Longstanding pain is common among children and adolescents, with prevalence rates varying between 11 and 38% (1). Recent reports indicate that prevalence increases with age and the occurrence of chronic or recurrent pain is more often found in girls than boys (Roth-Isigkeit et al., 2005; Stanford et al., 2008; King et al., 2011). Headache, abdominal pain, back pain and musculoskeletal pain represent the most frequently reported types of chronic pain among children and adolescents (Stanford et al., 2008; King et al., 2011)- and a relatively large number of youths report pain from multiple locations (Hoftun et al., 2011; King et al., 2011). For many children and adolescents, medical strategies are often ineffective or insufficient to alleviate symptoms and increase functioning.

A subsample of patients is severely affected by chronic pain, demonstrating low levels of functioning and quality of life. Functioning is a broad construct that can be subdivided into several different dimensions, such as physical, social, and emotional functioning (i.e., depression) (McGrath et al., 2008; Zernikow et al., 2012). More specifically, the presence of chronic pain can interfere with functioning with regard to quality of life (Huguet and Miro, 2008), sleeping, eating, and ability to pursue hobbies, as well as lead to absence from school and inability to lead an active social life (Konijnenberg et al., 2005; Simons et al., 2010).

The relationship between pain and functioning in children with chronic pain is complex, and information regarding factors associated with reduced functioning is still relatively scarce. However, some studies exist. For example, pain in multiple locations is associated with more disability (Hoftun et al., 2011; Holm et al., 2012), and depressive symptoms have been shown to predict school impairment (Gauntlett-Gilbert and Eccleston, 2007; Logan et al., 2009).

Importantly, existing research suggest that the ability to manage pain, in addition to pain intensity *per se*, is critical to functioning (Kashikar-Zuck et al., 2011; Kaczynski et al., 2013). From a behavior analytic (i.e., learning theory) perspective, anticipation of pain may result in avoidance of activities, even when perceived as important. Over time, such negatively reinforced behavior patterns, characterized by avoidance of pain, may result in a lowered level of functioning, without a corresponding decrease in pain.

Recent developments within Cognitive Behavior Therapy (CBT), particularly Acceptance and Commitment Therapy (ACT), has suggested the utility of pain management strategies based on acceptance and mindfulness to increase functioning (Wicksell et al., 2007, 2009). The treatment objective in ACT is to increase the ability to act in accordance with values and goals, also in the presence of interfering pain and distress (Hayes et al., 2006). In other words, treatment is not primarily aimed at reducing pain, but at reducing the impact of symptoms on behavior, i.e., pain interference. Thus, ACT and similar treatments may be particularly useful for a subgroup of individuals with avoidance and pain interference that result in low levels of functioning. However, more research is needed regarding factors (e.g., demographics, pain, pain interference) that characterize pediatric patients with chronic pain and low levels of functioning, and to explore the importance of these factors for the relation between pain and functioning.

Also, previous analysis have indicated that pain interference, as assessed by the pain interference index (PII), is tightly linked to pain intensity as well as functioning (Holmstrom et al., 2015). What distinguishes PII from other measures of functioning is that the PII was designed to specifically address the impact of pain on functioning, i.e., pain-related interference, whereas broader measures of functioning often take into account several different factors that can influence functioning, such as, developmental, social, and somatic problems other than pain. The scale includes questions such as; To what degree during the past 2 weeks has pain made it difficult for you to do schoolwork? Furthermore, the PII has been shown to independently predict variability in functioning above and beyond pain intensity (Holmstrom et al., 2015). Thus, the role of pain interference in the relation between pain and functioning should be further explored.

The purpose of the present study was to identify factors of importance for the relation between symptoms and disability. More specifically, the aims of the present study were to: (1) investigate if pediatric patients with chronic pain and high and low levels of functioning differ in demographic variables, pain, and pain interference; (2) explore the mediating function of pain interference in the relationship between pain and functioning (i.e., depression and functional disability).

## Materials and Methods

### Procedure and Participants

The study sample consisted of 163 consecutively recruited pediatric patients and their parents, referred to a tertiary pain clinic due to longstanding pain. Some data from this sample have been published previously in a paper addressing insomnia in children with chronic pain and as part of the validation of the PII (Kanstrup et al., 2014; Holmstrom et al., 2015). Both parent and child gave informed written consent and the study was approved by the Regional Ethical Review Board in Stockholm.

Self-report questionnaires were administered just prior to a medical and psychological assessment. All patients between 7 and 18 years and with sufficient Swedish language skills referred to the clinic between June 2008 and October 2011 due to longstanding and/or recurrent pain (i.e., >3 months) were considered eligible for participation. Very few families (<5) declined participation, and statistical analyses of differences in characteristic are therefore not considered meaningful.

### Assessments

The medical and psychological assessments consisted of two semi-structured clinical interviews conducted by a physician specialized in pediatric pain and a clinical psychologist trained in CBT or by self-report questionnaires (patients and parents) administered in conjunction with the interviews.

#### Interviews

Assessments focused on pain characteristics (e.g., pain intensity, location, and onset/duration), as well as the effects of pain on emotional, social, and physical functioning. For the present study, the following data were retrieved from the semi-structured interviews: (1) pain duration in months; (2) number of pain locations; (3) pain location/type, categorized as headache, abdominal pain, back pain, joint pain, complex regional pain syndrome (CRPS), wide spread pain (WSP) or other; (4) temporal pain patterns, categorized as continuous, daily, weekly, and monthly; (5) current school absence due to pain during the past month, classified as no absence (0), a few days of absence/month (1), >1 day/week of absence, (2), complete absence (3).

#### Pain Assessment

Current pain intensity, i.e., the patient’s subjective amount of experienced pain at that particular moment (i.e., total amount of pain during the interview), was rated on a numerical rating scale (NRS) from 0 to 10, where 0 = no pain and 10 = the worst imaginable pain (von Baeyer, 2009). The NRS is validated for pediatric samples 8 years and older (Miro et al., 2009; von Baeyer et al., 2009). A measure of current pain intensity was used in the present study since retrospective ratings have been reported to show inflated rates in children and adolescents (Lewandowski et al., 2009).

#### Center for Epidemiological Studies-Depression Scale Children (CES-DC)

Symptoms of depression during the past week were measured by the Center for Epidemiological Studies-Depression Scale Children (CES-DC). The questionnaire consists of 20-items that are rated on a scale from 0 (not at all) to 3 (often), with a maximum score of 60. The Swedish version of the scale, with a high reliability coefficient alpha (0.91) and validated for children (6 years and older) and adolescents with a cut-off score of 24 as an indicator of major depression, was used in the present study (Fendrich et al., 1990; Olsson and von Knorring, 1997).

#### Functional Disability Inventory-Parent version (FDI-P)

This instrument comprises 15 questions regarding functioning in everyday activities, rated on a scale from 0 (no problems) to 4 (impossible). The maximum score is 60 and suggested cut-offs are; 0–12 (no disability), 13–20 (mild), 21–29 (moderate) and >30 (severe disability). Reports on the FDI-P has shown good correspondence between parent and child ratings in addition to satisfactory validity and reliability (Walker and Greene, 1991; Claar and Walker, 2006).

#### Pain Interference Index (PII)

The PII was developed as a brief instrument to specifically address pain related interference in everyday life. The Swedish version of the PII used in the present study has showed adequate statistical properties in a sample of children and adolescents 7–18 years (Holmstrom et al., 2015). Also, an English version of PII, including a parent version of the instrument, has recently been validated based on a sample of patients with neurofibromatosis aged 6–25 years (Martin et al., 2015). PII consist of six items rated on a scale from 0 (not at all) to 6 (very high) with a maximum total score of 36. The child is asked to what degree during the past 2 weeks pain has: (1) Made it difficult for you to do schoolwork, (2) Made it difficult for you to do activities outside school (leisure activities), (3) Made it difficult for you to spend time with friends, (4) Affected your mood, (5) Affected your ability to do physical activities (like run, walk upstairs, play sports), and (6) Affected your sleep.

### Statistical Analyses

#### Patient Characteristics

Descriptive statistics were used to summarize sample characteristics (age, sex, pain locations, temporal pain pattern, pain duration over time, current pain intensity, and school absence). Student’s *t*-tests were used to compare means between subgroups. Zero-order correlations were investigated with Pearson’s *r* and internal consistency were investigated with Cronbach’s alpha. Data is presented for the whole group, as well as divided into males and females.

#### Mediation Analyses

All analyses were conducted using SPSS version 22.

To explore the importance of pain interference for the relationships between pain and functioning (i.e., depression and functional disability), a mediation model was tested with pain intensity as the independent variable (X), PII as the mediator (M), and CES-DC or FDI-p as the dependent variables (Y). The product of coefficients approach was used, which is today widely viewed as the best overall test of mediation (MacKinnon et al., 2007). Also, although the Normal theory test may be used to assess the indirect effects of pain interference on the relationships between pain and functioning, recent methods have advocated bootstrapping, a non-parametric resampling procedure (Preacher and Hayes, 2004). In the present study, results from both the Normal theory test (parametric) and the bootstrapping approach (non-parametric) are presented. Furthermore, analyses were conducted to address the issue of directionality (i.e., if the functional relationship between M and Y variables is opposite to what is defined *a priori*). Specifically, the dependent variables (depression, functional disability) were entered into the analytic model as mediators, while the proposed mediator (pain interference) was used as dependent variable, essentially inverting the original analyses. Missing values were excluded listwise in all analysis. An α-level of *p* < 0.05 was chosen as threshold for statistical significance and two-tailed tests were used in all analyses.

Analyses of mediators should be based on theoretically relevant *a priori* hypotheses. In the present study, a conceptual model based on a behavioral analytic framework is tested. It is well known that chronic pain commonly results in disability, including reduced levels of physical, social, and emotional functioning. A wide variety of interventions exist to improve functioning, each with a more or less distinct treatment objective. For example, medical strategies are typically aimed at reducing pain intensity. In contrast, behavioral interventions such as ACT are not primarily aimed reducing pain but at reducing the impact of pain on behavior, i.e., pain interference. Thus, functioning may be increased by a reduction in pain interference, also when pain intensity remain relatively unchanged. This type of intervention is based on a conceptual model in which the relationship between pain and functioning is mediated by another, and modifiable, variable (i.e., pain interference). However, to our knowledge there are to date no studies that have evaluated the importance of pain interference as a mediator between pain and functioning in pediatric chronic pain. In the present study, it was hypothesized that pain interference mediates the relationship between pain intensity and depression, as well as between pain intensity and functional disability.

## Results

### Sample Characteristics

The mean age in this sample (*n* = 163) was 14.1 years (*SD* = 2.6), 121 girls (74.2%) were included in the sample.

A large proportion (75.3%) of the patients reported pain from multiple locations and the most frequently reported type of pain was headache (65.9%), while 40.6% reported stomach pain, 30.6% back pain and 22.9% pain from joints, 12% widespread pain and 12% were diagnosed with CRPS. Over 55% of total the sample reported to have continuous pain, and 22% reported episodes of pain on a daily basis.

The total sample mean for current pain intensity was 4.4 (*SD* = 2.8, range 0–10), with 15% (*n* = 23) of the sample reporting a pain intensity of >7. The mean pain duration in the total sample at the time for data collection was 51.4 months (*SD* = 43, range 3–192 months) or approximately 4 years. Current pain intensity was significantly correlated with the PII (*r* = 0.39, *p* < 0.01) and the CES-DC (*r* = 0.21, *p* < 0.01), but not with the FDI-P.

The mean score on the CES-DC for the total group was 23.1 (*SD* = 12.1), with 44% of the sample scoring above the suggested cut-off for major depression (see Materials and Methods). The mean score on FDI-P was 16.5 (*SD* = 11.7) for the total sample, indicative of overall mild disability according to suggested cut-offs (see Materials and Methods), and 15% of the sample had a score higher than the suggested cut-off for severe disability. The sample mean for pain interference (PII) was 18.3 (*SD* = 9.4) of a maximum 36. The PII correlated significantly with the CES-DC (*r* = 0.68, *p* < 0.01) and the FDI-P (*r* = 0.55, *p* < 0.01). There was also a significant but weaker relationship between the CES-DC and FDI-P (*r* = 0.33, *p* < 0.01). The internal consistency of the scales, as measured by Cronbach’s alpha, was found to be high in the present sample, 0.82 for the CES-DC, 0.86 for the PII and 0.91 for the FDI-P.

School absence due to pain was frequently reported within the sample, with over 70% of the patients staying home from school or missing classes due to pain at least once a week. Also, 13% of the children/adolescents that reported school absence due to pain were not attending school at all (**Table 1**).

**Table 1:** Pain related school absence.

Pain related school absence (*N* = 161)	Frequency	Percent
No absence	43	26.7
A few days of absence/month	49	30.4
>1 day/week of absence	48	29.8
Complete absence	21	13
Total	161	100

### Differences in Pain and Functioning between Subgroups of Patients

Subgroups of patients based on gender and number of pain locations were compared to evaluate possible differences in age, pain (intensity, duration and interference) and functioning (functional disability, depression).

#### Gender

In this sample, girls experienced significantly more depression than boys. In contrast, boys illustrated longer pain duration than girls, however, this difference did not reach statistical significance. No significant differences were found between boys and girls in pain intensity, disability or pain interference, see **Table 2**.

**Table 2:** Pain and functioning in subgroups of patients.

	Gender			Multiple pain locations			CES-DC			FDI-P		
	Girls (*N* = 121)	Boys (*N* = 42)	*p*	Single (*N* = 41)	Multiple (*N* = 122)	*p*	Low (*N* = 85)	High (*N* = 78)	*p*	Low (*N* = 138)	High (*N* = 25)	*p*
Age (years) *M* (*SD*)	14.3 (2.5)	13.5 (2.9)	0.09	13.78 (3.4)	14.2 (2.4)	0.42	13.3 (2.9)	14.9 (2.1)	0.00^∗^	13.9 (2.7)	14.9 (2.1)	0.8
Pain duration *M* (*SD*)	47.0 (41.2)	63.9 (46.0)	0.04	40.3 (38.2)	55.3.2 (44.2)	0.06	51.9 (39.7)	50.9 (46.6)	0.89	51.8 (42.6)	49.2 (45.8)	0.02
Pain (NRS) *M* (*SD*)	4.5 (2.7)	4.0 (3.2)	0.34	3.8 (3.0)	4.6 (2.8)	0.115	3.8 (2.8)	4.9 (2.9)	0.02	4.1 (2.8)	5.6 (3.1)	0.80
PII *M* (*SD*)	19.2 (9.3)	16.1 (9.5)	0.09	17.2 (11.9)	18.7 (8.8)	0.35	13.0 (8.0)	24.1 (7.3)	0.00^∗^	17.0 (9.3)	25.2 (7.1)	0.00^∗^
CES-DC *M* (*SD*)	24.7 (12.2)	18.3 (10.3)	0.00^∗^	21.7 (12.0)	23.6 (12.1)	0.45	13.6 (6.3)	33.4 (7.4)	0.00^∗^	22.0 (12.2)	29.3 (9.6)	0.00^∗^
FDI-P *M* (*SD*)	16.6 (11.4)	16.5 (12.8)	0.96	14.9 (13.5)	17.1 (11.2)	0.26	13.3 (10.8)	19.9 (11.8)	0.00^∗^	12.7 (8.3)	36.1 (6.6)	0.00^∗^

*^∗^Significant at the *p* < 0.01 level. M, Mean; SD, Standard deviation; N, Number of subjects; NRS, numerical rating scale. Pain duration is presented in months. PII, Pain Interference Index; CES-DC, Center for Epidemiological Studies-Depression Scale Children; FDI-P, Functional Disability Inventory-Parent version. Low = below cut off; High = above cut off.*

#### Single or Multiple Pain Locations

Children with pain from multiple locations (*n* = 122) were compared to the group of children with pain from a single location (*n* = 41). No significant differences between these two subgroups were found, showing that children/adolescents with pain from multiple sites were not more impaired (as measured by PII, CES-DC, and FDI-P), not experiencing higher levels of pain and had not been experiencing pain for a longer period of time, see **Table 2**.

#### Comparing Patients With and Without Depression

A series of analyses were conducted to compare patients with scores above and below the cut-off for major depression (24) on age, pain intensity, pain duration, pain interference, and functional disability. Patients with a score indicative of major depression (>23, *n* = 78) had significantly higher scores on the PII and FDI-P and were significantly older when compared to the patients with CES-DC scores below the suggested cut off. However, no significant difference could be found between subgroups with higher/lower depression scores regarding pain duration, see **Table 2**.

#### Functional Disability

Similarly, a subgroup analysis was carried out to compare patients (*n* = 25) scoring above and below the suggested cut-off for severe disability on the (FDI-P > 30). The subgroup with severe disability displayed significantly higher levels of depression and pain interference, compared to patients with lower scores on disability (i.e., no disability to moderate disability. There were no significant differences in age, pain duration, or pain intensity between the disability subgroups.

### Pain Interference as a Mediator between Pain and Functioning

**FIGURE 1:**
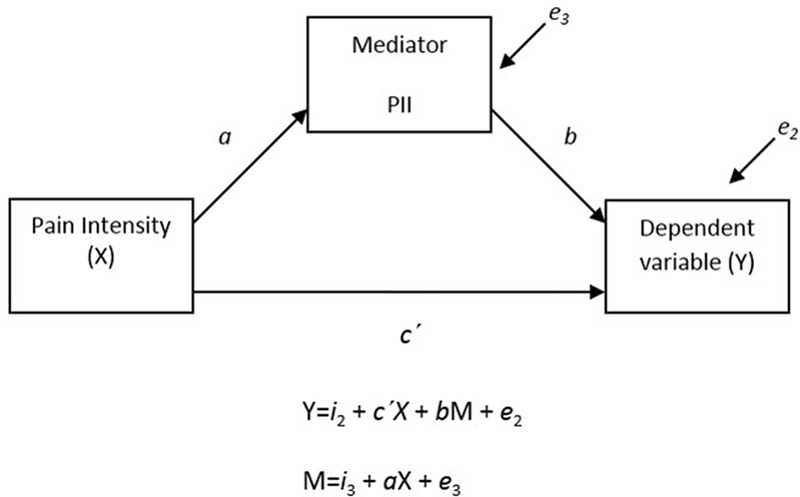
**Mediation analysis; The influence of pain interference on the relation between pain and functioning was evaluated by analyzing the indirect effect of PII in the association of (1) pain intensity (X) and CES-DC (Y), and (2) pain intensity (X) and FDI-P (Y)**.

The influence of pain interference on the relation between pain and functioning was evaluated by analyzing the indirect effect of PII in the association of (1) pain intensity and CES-DC, and (2) pain intensity and FDI-p (**Figure 1**).

#### The Relation between Pain and Depression

Significant indirect effects (*p* < 0.01) of pain interference in the relationship between pain intensity and depression was seen in the Normal Theory Test as well as when using a bootstrap approach. The Normal Theory Tests revealed that both the a and b paths were significant. Furthermore, the relation between the predictor (pain) and outcome variable (depression) changed from significant to non-significant when controlling for the indirect effects (mediator), suggesting that the relationship between pain and depression is strongly influenced by the pain interference.

#### The Relation between Pain and Functional Disability

Consistent with the findings on depression, both the Normal Theory Test and the bootstrap method illustrated a significant indirect effect (*p* < 0.01) of pain interference on the relation between pain and functional disability. Results are summarized in **Table 3**.

**Table 3:** The mediating role of pain interference in the relationships between pain intensity and depression, as well as between pain intensity and functional disability.

The effects of pain interference on the relation between pain and depression				
Normal theory test				
Path	Coefficient	*SE*	*t*^a^	*p*
*a*	1.28	0.25	5.14	<0.0001
*B*	0.90	0.08	11.05	<0.0001
Total *(c)*	0.90	0.33	2.68	0.0081
Direct *(c′)*	-0.25	0.27	-0.92	0.3602
*a^∗^b*	1.14	0.24	4.68	<0.0001

**Non-parametric bootstrap approach**				

			**CI (95%)^b^**	
**Mediator**	**Mean indirect effect**	***SE***	**Lower**	**Upper**

Depression	1.14	0.23	0.60	1.78

**The effects of pain interference on the relation between pain and functional disability**				

**Normal theory test**				

**Path**	**Coefficient**	***SE***	***t*^a^**	***p***

1-5 *a*	1.29	0.26	4.97	<0.0001
*B*	0.70	0.09	7.63	<0.0001
Total *(c)*	0.66	0.34	1.95	0.0533
Direct *(c′)*	-0.24	0.31	-0.77	0.4444
*a^∗^b*	0.90	0.22	4.18	<0.0001

**Non-parametric bootstrap approach**				

			**CI (95%)^b^**	
**Mediator**	**Mean indirect effect**	***SE***	**Lower**	**Upper**

Functional disability	0.90	0.20	0.42	1.47

#### Examining Directionality

To examine the issue of directionality, two analyses were performed with each of the dependent variables (depression or functional disability) entered as mediator of the relation between pain intensity and pain interference (essentially reversing the original mediation analyses). Neither of these results were significant, providing incremental yet tentative support for the directionality of the meditational effect illustrated in the original analyses.

## Discussion

An increasing number of studies have illustrated that chronic pain is commonly associated with low levels of functioning. However, little is yet known about how specific factors influence the complex interplay between pain and functioning. To investigate if pediatric chronic pain patients with high and low levels of functioning differed in demographic variables, pain, and pain interference and to explore the mediating function of pain interference in the relationship between pain and functioning (i.e., depression and functional disability) a series of analysis was carried out in a sample of pediatric patients referred to a tertiary care pain clinic.

Findings from the present study showed that, older participants presented with higher levels of depression and pain interference, corresponding with a previous study showing that decreased functioning in daily life may be related to age (Roth-Isigkeit et al., 2005). In line with previous research (Piccinelli and Wilkinson, 2000), girls reported higher levels of depression. However, girls and boys reported similar levels of pain intensity, pain interference and disability. In contrast to previous studies reporting that pain in multiple locations is associated with more severe disability, participants with pain from multiple sites did not demonstrate higher levels of pain, pain interference, disability or depression than those experiencing pain from a single location in the present sample (Hoftun et al., 2011; Holm et al., 2012) and the patients displaying the most impaired functioning (depression, high pain interference and decreased physical functioning) were not the patients that had experienced pain over the longest period of time, nor where they the patients that were experiencing the highest levels of pain.

The association between pain and depression is well established in adults, and this study provides further support that these variables are strongly correlated also in youths with chronic pain. Scores above the suggested cut-offs for depression were found in almost half of the total sample, with a mean score on the depression measure significantly higher in girls compared to boys. These findings further emphasize the close relationship between chronic pain and depression found in several recent studies (Claar and Walker, 2006; Zernikow et al., 2012).

Previous research has shown that the relationship between pain intensity and functioning is less direct than expected (Claar and Walker, 2006), pointing at a need to further explore how these and other related variables are associated. It can be argued that pain interference is a critical factor in the development of depression in youths with chronic pain. The avoidance of physical and social activities that are perceived as meaningful although associated with pain may reduce pain and distress in the short run, but may over time result in a less active and meaningful life. Results from the present study indicated that pain interference is a key factor in the complex relationship between pain and functioning. Although tentative due to the cross-sectional data set, results from the present study suggest that the mediating role of pain interference should be further evaluated in longitudinal studies and clinical trials. Thus, the present findings support the notion that pain interference might be a more important factor in relation to functioning than levels or duration of pain. This is line with recent research, emphasizing the need for a shift in focus to the behavioral aspect of pain (Palermo, 2009). It is of utmost importance to adequately capture the impact of chronic pain in children, and the present findings suggests that pain interference is a highly relevant dimension. In addition, the alarming prevalence of chronic debilitating pain calls for further development of interventions that reduce pain interference among children and adolescents where symptoms may remain, such as CBT and ACT (24).

Although the empirical support for this type of treatment is relatively strong, more research is needed to clarify individual characteristics of treatment responders, particularly in pediatric chronic pain. For example, it is possible that patient characteristics (i.e., age, pain duration) moderate the effects of treatment. If we can identify patient characteristics (e.g., demographics, pain, pain interference, depression) of individuals with low levels of functioning, this will improve the ability to tailor treatment to meet the individual needs of each patient which may improve effect sizes.

A number of limitations should be taken into account when interpreting the results from this study. It should be noted that the sample in this study was selected on the basis of referral to a tertiary pain clinic and it is thus possible that the included children and youths represent a sub group of individuals that are particularly affected by their chronic pain. The use of cross-sectional data obviously prevents any causal conclusions. Furthermore, it is important to emphasize, that a cross sectional design only provides a pattern of results that suggest the importance of pain interference for the relationship between, e.g., symptoms and depression. Longitudinal studies are needed to confirm these findings in addition to studies investigating the relative importance of different hypothesized mediators. Although the child and parent version of the FDI has shown to correlate well, it is possible that the use of the child version had provided different results on disability, and it may be argued that including both versions would have facilitated a relevant comparison between parent and child reports, as well as between PII and FDI. In addition, more information regarding pain, e.g., average pain intensity over the past weeks, would have been useful to validate the correlations between, e.g., pain and depression. Also, it would have been desirable to have data on the current pain management of the included children since this could have added another dimension to the findings, however, this was not assessed in a structured way in the present study. Furthermore, data for the present study was collected in clinical interviews or by self-report questionnaires. Thus the present study used self-reports only and it is suggested that future studies include objective measures of functioning, such as actigraphic monitoring or records of school absence provided by teachers and the results in the present study should be cross-validated in a study with a different, and ideally larger, sample.

## Conclusion

Thus, this study adds to the growing support of findings suggesting that functioning and pain interference should be routinely assessed in pediatric chronic pain and a central target in treatment. Particularly, these findings imply a need for interventions specifically aimed at improved functioning for patients with chronic debilitating pain.

## Ethics Statement

The study sample consisted of 163 consecutive pediatric patients and their parents, referred to a tertiary pain clinic due to longstanding pain. Both parent and child gave informed written consent and the study was approved by the local ethics committee. A written copy of the study information sheet was given to the families when arriving at the hospital. Families were given sufficient time to read the information and ask any question they might have. Consent was then obtained prior to data collection (at the first visit to the clinic). Both parents and child were given written and oral information prior to accepting the invitation to partake in the study, and informed consent was obtained from both parties.

## Author Contributions

LH has been responsible for the study design, analysis and manuscript preparation (in collaboration with RW). RW has been responsible for the study design, analysis and manuscript preparation (in collaboration with LH). MaK has been responsible for data collection and setting up the data base, taken part in analysis and have commented on the manuscript through out the writing process. MiK has taken part in study design, data collection and analysis and have commented on the manuscript through out the writing process.

## Conflict of Interest Statement

The authors declare that the research was conducted in the absence of any commercial or financial relationships that could be construed as a potential conflict of interest.
